# Fluoride-Incorporated Apatite Coating on Collagen Sponge as a Carrier for Basic Fibroblast Growth Factor

**DOI:** 10.3390/ijms25031495

**Published:** 2024-01-25

**Authors:** Aniruddha Pal, Ayako Oyane, Maki Nakamura, Kenji Koga, Erika Nishida, Hirofumi Miyaji

**Affiliations:** 1 Nanomaterials Research Institute, National Institute of Advanced Industrial Science and Technology (AIST), AIST Tsukuba Central 5, 1-1-1 Higashi, Tsukuba 305-8565, Japan; pal-aniruddhapal8@aist.go.jp (A.P.); ma-ki-nakamura@aist.go.jp (M.N.); k.koga@aist.go.jp (K.K.); 2 Section for Clinical Education, Faculty of Dental Medicine, Hokkaido University, N13 W7 Kita-ku, Sapporo 060-8586, Japan; erikanishida@den.hokudai.ac.jp (E.N.); miyaji@den.hokudai.ac.jp (H.M.)

**Keywords:** apatite, collagen, basic fibroblast growth factor (bFGF), tailored release, scaffolds, fluoride

## Abstract

Coating layers consisting of a crystalline apatite matrix with immobilized basic fibroblast growth factor (bFGF) can release bFGF, thereby enhancing bone regeneration depending on their bFGF content. We hypothesized that the incorporation of fluoride ions into apatite crystals would enable the tailored release of bFGF from the coating layer depending on the layer’s fluoride content. In the present study, coating layers consisting of fluoride-incorporated apatite (FAp) crystals with immobilized bFGF were coated on a porous collagen sponge by a precursor-assisted biomimetic process using supersaturated calcium phosphate solutions with various fluoride concentrations. The fluoride content in the coating layer increased with the increasing fluoride concentration of the supersaturated solution. The increased fluoride content in the coating layer reduced its solubility and suppressed the burst release of bFGF from the coated sponge into a physiological salt solution. The bFGF release was caused by the partial dissolution of the coating layer and, thus, accompanied by the fluoride release. The concentrations of released bFGF and fluoride were controlled within the estimated effective ranges in enhancing bone regeneration. These findings provide useful design guidelines for the construction of a mineralized, bFGF-releasing collagen scaffold that would be beneficial for bone tissue engineering, although further in vitro and in vivo studies are warranted.

## 1. Introduction

Bone tissue engineering has continued to advance since the concept of “tissue engineering” was first proposed almost three decades ago. Scaffolds with a 3D porous structure that facilitates cell recruitment and nutrient flow are often used in tissue engineering [1,2]. Human bone tissue is composed of collagen nanofibers with low-crystalline, non-stoichiometric hydroxyapatite (called apatite for short) nanocrystals deposited on their surfaces [3,4]. To date, various approaches to modify collagen-based 3D scaffolds with apatite or other osteoconductive materials have been proposed to develop a scaffold for bone tissue engineering [4,5,6,7,8,9,10,11,12,13,14]. The proposed approaches include biomimetic coating [5,6], coprecipitation [7,8], particle blending [9,10,11], alternate dipping [12], electrospinning [13], and 3D printing [14]. Among these, the biomimetic coating approach is relatively time consuming. However, it has several advantages. For example, it can produce bone-like apatite under mild reaction conditions without using any toxic reagents and coat the entire surface of a porous collagen scaffold, thereby toughening the scaffold (which is beneficial for the retention of its porous structure). Furthermore, the biomimetically coated apatite layers can be loaded with a variety of bioactive substances, such as proteins, trace elements, and nucleic acids [6,15,16,17,18].

The present authors recently developed a 3D porous scaffold with a mineralized surface by coating low-crystalline apatite on a collagen sponge using a precursor-assisted biomimetic process [5]. In this process, a collagen sponge is subjected to oxygen plasma treatment to increase the water wettability of its porous surface. The plasma-treated sponge is immersed in a calcium solution to allow the adsorption of calcium ions and then immersed in a phosphate solution to precoat the surface with amorphous-phase calcium phosphate (CaP). The CaP-precoated sponge is subsequently immersed in a supersaturated CaP solution, in which the precoated CaP acts as a precursor for apatite growth. The resulting apatite-coated collagen sponge showed superior functionality as a scaffold for bone tissue engineering compared with the uncoated sponge, exhibiting enhanced cellular ingrowth in rat subcutaneous tissue [5], and promoted bone regeneration in a rat cranial bone defect model while being resorbed and replaced with regenerated bone tissue [19].

The functionality of the apatite-coated collagen sponge should improve further if the apatite layer could be loaded with basic fibroblast growth factor (bFGF), a clinically approved therapeutic protein that enhances the regeneration of both soft and hard tissues [20,21,22]. To date, bFGF-immobilized apatite layers have been coated on artificial materials, including metallic implants [23], polymeric substrates [24], and ceramic disks [25,26] using biomimetic processes (not yet on collagen sponges, however). The layers have been shown to release bFGF under physiological conditions [24,25,26] and promote angiogenesis [26], bone regeneration [23,25], and osteointegration [23]. In these previous studies, the tuning of bFGF release was achieved mainly by varying the bFGF content in the coating layers. Here, we propose another approach focusing on the apatite phase, a matrix of the coating layer. We hypothesized that the incorporation of fluoride ions into apatite crystals would enable the tailored release of bFGF from the coating layer, depending on the layer’s fluoride content. This hypothesis was based on the previous reports that biomolecule-immobilized apatite layers can release immobilized biomolecules through the partial dissolution of the apatite crystals [27] and the incorporation of fluoride into apatite crystals (via ionic substitution for hydroxyl ions) reduces their dissolution rate by decreasing their aqueous solubility [28,29].

The purpose of this study was to fabricate collagen sponges coated with layers consisting of fluoride-incorporated apatite (FAp) crystals with immobilized bFGF (bFGF-immobilized FAp layers) with varying fluoride contents using a precursor-assisted biomimetic process and to demonstrate the tailored release of bFGF from the coated sponges. In the precursor-assisted biomimetic process, supersaturated CaP solutions supplemented with bFGF and NaF were used as a growth medium for the coating layer. The fluoride concentration in the CaP solution was varied in order to alter the fluoride content of the resulting coating layer. The coating layers were characterized, and bFGF-release was assayed using a physiological salt solution.

## 2. Results

### 2.1. Surface Characterization

The collagen sponges (3 mm thickness, 5 mm diameter) were subjected to the same precursor-assisted biomimetic process for surface coating as previously described [5], with slight modifications using CaP solutions with various bFGF and NaF concentrations (for details, see Section 4.1, Section 4.2 and Section 4.3). The surface-coated sponge prepared using the CaP solution with neither bFGF nor NaF was designated as FGF0F0, and those prepared using the bFGF-containing CaP solutions with NaF concentrations of 0, 10, 100, and 1000 μM were designated as FGF10F0, FGF10F10, FGF10F100, and FGF10F1000, respectively. The untreated and coated sponges were cut in half horizontally (two disks of ~1.5 mm in thickness were prepared from one sponge) with a microtome blade to expose their interior surfaces (hereafter referred to as inner surfaces).

**Figure 1:**
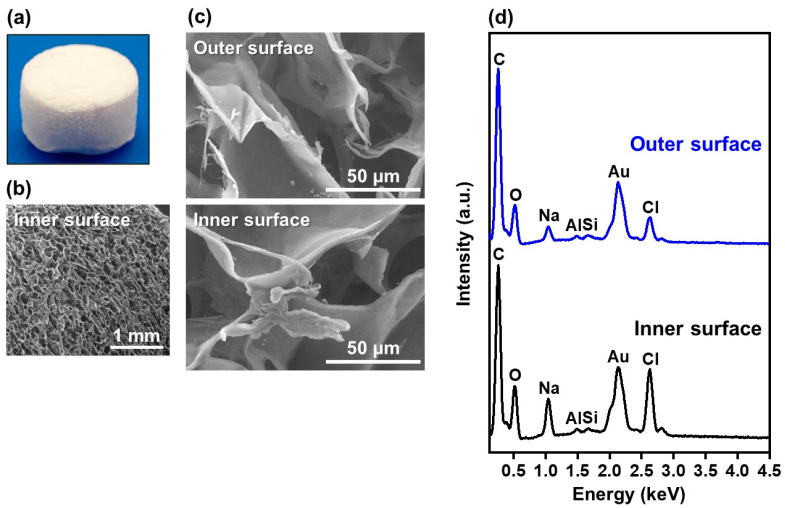
(**a**) Digital image of the untreated collagen sponge and (**b**,**c**) SEM images and (**d**) SEM-EDX spectra of its outer and inner surfaces. The peaks of Al and Au in (**d**) are attributed to an aluminum sample holder and a conductive coating for observation, respectively.

**Figure 2:**
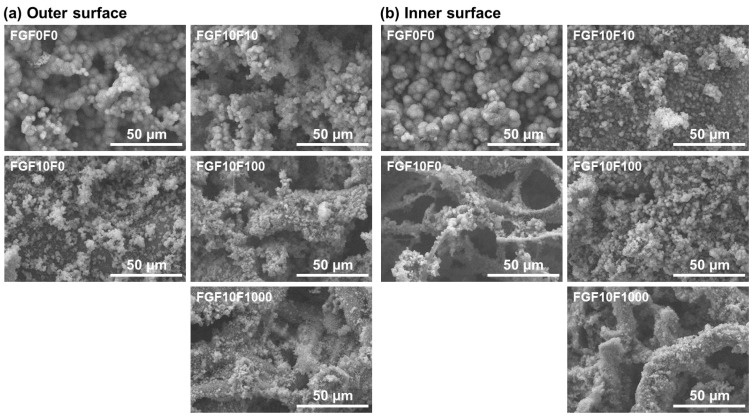
SEM images of the (**a**) outer and (**b**) inner surfaces of FGF0F0, FGF10F0, FGF10F10, FGF10F100, and FGF10F1000.

**Figure 3:**
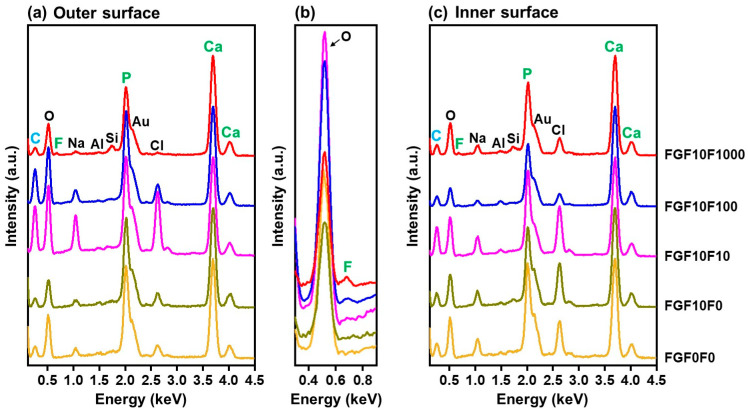
SEM-EDX spectra of the (**a**,**b**) outer and (**c**) inner surfaces of FGF0F0, FGF10F0, FGF10F10, FGF10F100, and FGF10F1000. (**b**) Narrow region spectra of (**a**). The peaks of Al and Au in (**a**,**c**) are attributed to an aluminum sample holder and a conductive coating for observation, respectively.

Both the outer and inner surfaces of the collagen sponges were coated with fluoride-incorporated CaP layers using the present precursor-assisted biomimetic process. Scanning electron microscope (SEM) images clearly indicated the porous structure of the untreated sponge (Figure 1a–c). On its outer and inner surfaces, Na, Si, and Cl were detected in addition to the component elements of collagen (C and O) by an energy-dispersive X-ray (EDX) spectrometer in SEM (Figure 1d), suggesting the presence of sodium chloride (NaCl) and Si-containing compounds on the untreated collagen sponge. These compounds are considered to be residues in the production process of the collagen sponge. After the precursor-assisted biomimetic process, submicro- or micro-scale particulate layers appeared on the outer and inner surfaces of the sponges, irrespective of types of CaP solutions (Figure 2). These layers were composed primarily of CaP, as evidenced by strong Ca and P peaks and the reduced intensity of the C peak (the primary component element of collagen) in the SEM-EDX spectra (Figure 3a,c). In the SEM-EDX spectra of FGF10F100 and FGF10F1000, a small peak for F was detected (Figure 3b), indicating that the CaP layers on their surfaces incorporated fluoride ions. In the SEM-EDX spectrum of FGF10F10, the F peak was not clearly detected. The peak intensity of F with respect to that of Ca appeared to increase with the increasing fluoride concentration in the CaP solution from 10 to 1000 µM (FGF10F10 < FGF10F100 < FGF10F1000).

**Figure 4:**
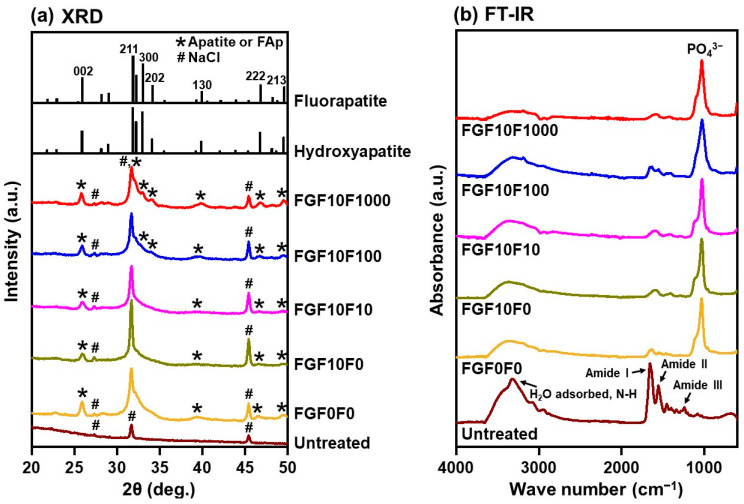
(**a**) XRD patterns and (**b**) FT-IR spectra of the untreated collagen sponge and FGF0F0, FGF10F0, FGF10F10, FGF10F100, and FGF10F1000. The reference data of hydroxyapatite and fluorapatite in (**a**) are from [30,34], respectively.

The X-ray diffraction (XRD) analysis revealed that the crystalline phase of the CaP layers on the collagen sponges was apatite or FAp. The XRD pattern of the untreated collagen sponge showed diffraction peaks ascribed to NaCl (Figure 4a). Besides these peaks, peaks ascribed to apatite or FAp were detected for the coated sponges, and the intensity of these peaks increased with the increasing fluoride concentration of the CaP solution from 10 to 1000 µM (FGF10F10 < FGF10F100 < FGF10F1000). The apatite 130 peak position of FGF0F0 (39.4°) shifted to 39.6° in FGF10F100 and 39.9° in FGF10F1000 and became closer to that of fluorapatite (39.9° in [30] and 40.2° in [31]). This peak shift should be owing to the partial substitution for hydroxyl ions in hydroxyapatite with fluoride ions [31]. Other CaP phases, such as octacalcium phosphate, were not detected in any sponges (XRD patterns with the 2θ region from 3 to 50° were checked). These results confirmed that all of the collagen sponges prepared using the present precursor-assisted biomimetic process were coated with apatite- or FAp-based layers. This conclusion was further supported by Fourier-transform infrared (FT-IR) spectrometry. The FT-IR spectrum of the untreated collagen sponge showed peaks ascribed to adsorbed H_2_O, N-H, amide I, amide II, and amide III bands [32,33] (Figure 4b). These peaks from collagen decreased in intensity, and a strong PO_4_^3−^ peak attributed to apatite or FAp was observed in the FT-IR spectra of the coated sponges.

### 2.2. bFGF Content in the Coated Sponges

**Figure 5:**
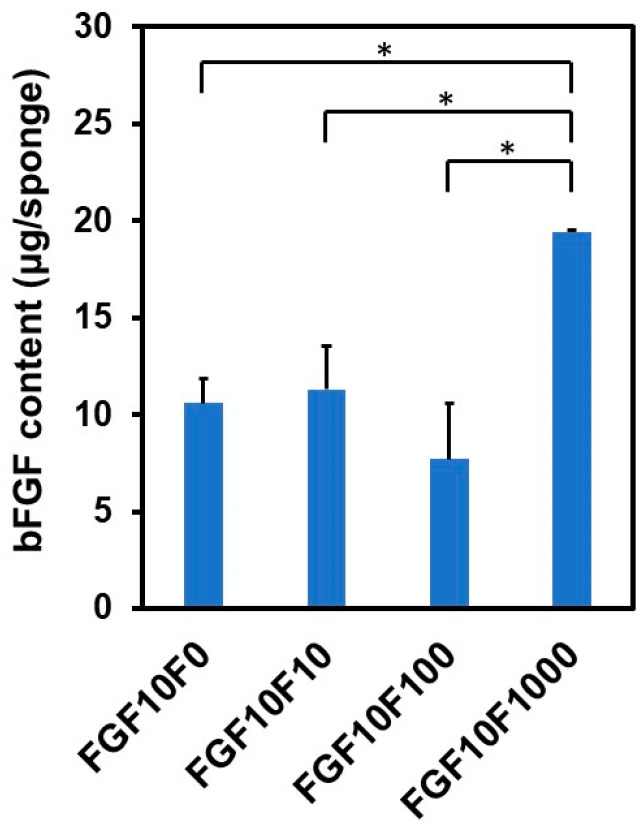
Content of bFGF immobilized in FGF10F0, FGF10F10, FGF10F100, and FGF10F1000 (*n* = 3, average + standard deviation (SD), * *p* < 0.05).

All of the coated sponges, except for FGF0F0, immobilized bFGF, according to the results of the Bradford protein assay. Figure 5 shows the amount of bFGF immobilized in FGF10F0, FGF10F10, FGF10F100, and FGF10F1000. The bFGF content was higher in FGF10F1000 than in the other sponges.

### 2.3. Detailed Analysis of FGF10F0 and FGF10F1000

**Figure 6:**
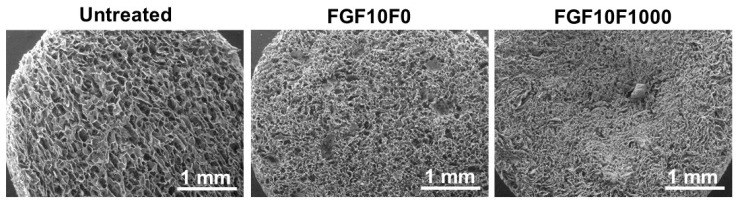
SEM images of the inner surfaces of the untreated collagen sponge, FGF10F0, and FGF10F1000.

Fluoride incorporation into the coating layer affected the porous structure of the coated sponge. The selected sponges (FGF10F0 and FGF10F1000) were subjected to detailed analyses. Cross-sectional SEM observation clarified that the porosity of the sponges apparently decreased in the following order: untreated sponge > FGF10F0 > FGF10F1000 (Figure 6). This observation was supported by 2D porosity measurements using a laser microscope, in which the 2D porosity of the untreated sponge, FGF10F0, and FGF10F1000 was 58 ± 10%, 44 ± 6%, and 38 ± 6%, respectively (Table 1). The difference between the untreated sponge and FGF10F1000 was statistically significant (*p* = 0.039), although there was no significant difference between the untreated sponge and FGF10F0 (*p* = 0.108) or between FGF10F0 and FGF10F1000 (*p* = 0.259). As verified by weight measurements, the coating weight of FGF10F1000 was significantly heavier (*p* = 0.012) than that of FGF10F10 (Table 1), suggesting a thicker coating layer in the former. This result corresponds to the XRD results shown in Figure 4a.

**Figure 7:**
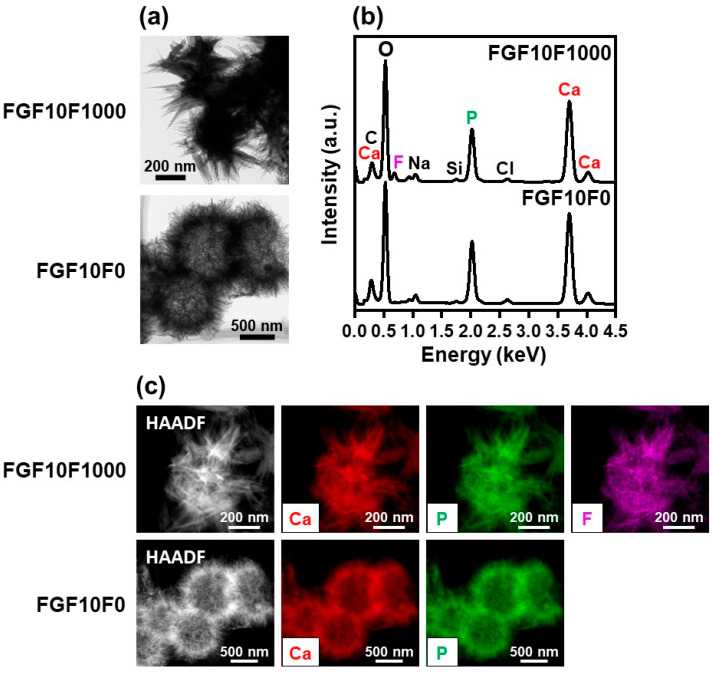
(**a**) TEM images, (**b**) STEM-EDX spectra, and (**c**) high-angle annular dark-field (HAADF) images and corresponding STEM-EDX elemental (Ca, P, F) mapping images of FGF10F0 (**lower row**) and FGF10F1000 (**upper row**).

Fluoride incorporation into the coating layer altered the nanostructures as well as the composition of the crystals in the layer. Transmission electron microscope (TEM) observation revealed the fine needle-like structures of the apatite crystals in FGF10F0 (Figure 7a). The FAp crystals in FGF10F1000 were thicker, longer, and apparently more rigid than the apatite crystals in FGF10F0. Scanning TEM (STEM)-EDX analysis reconfirmed the SEM-EDX results shown in Figure 3, in which the FAp crystals in FGF10F1000 contained fluorine, whereas the apatite crystals in FGF10F0 did not (Figure 7b). As visualized with STEM-EDX elemental mapping images (Figure 7c), fluorine distributed homogeneously over the FAp crystals in FGF10F1000. The apatite crystals in FGF10F0 had a Ca/P molar ratio of 1.54 ± 0.02 (Table 1, calculated from the STEM-EDX results), suggesting that they are calcium-deficient apatite. The FAp crystals in FGF10F1000 had a higher Ca/P molar ratio of 1.65 ± 0.03, a ratio close to that (1.67) of stoichiometric apatite and fluorapatite: Ca_10_(PO_4_)_6_F_2_. The FAp crystals in FGF10F1000 had a F/P molar ratio of 0.14 ± 0.04, which was approximately 60% lower than that (0.33) of fluorapatite.

### 2.4. bFGF-Release Assay

**Figure 8:**
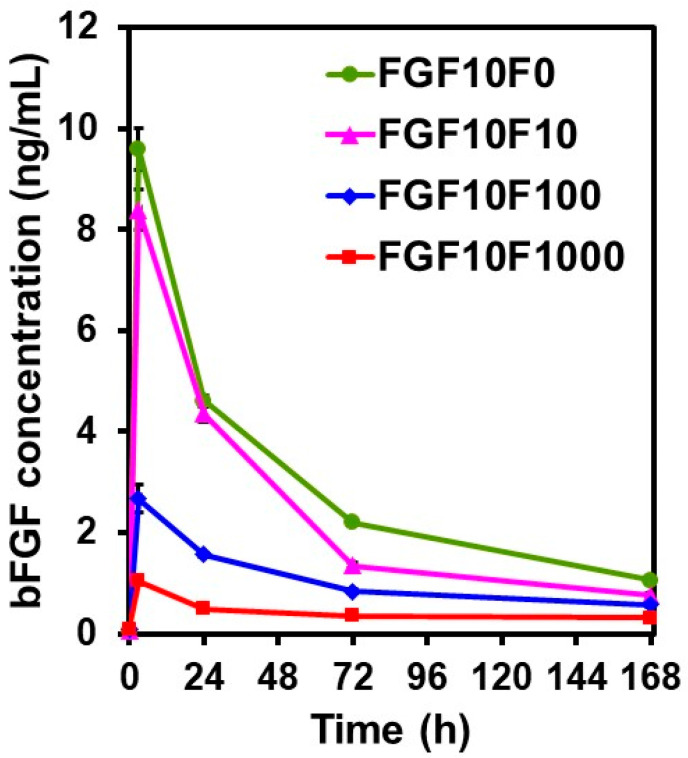
Change in bFGF concentration (measured by ELISA) in the physiological salt solution during incubation of FGF10F0, FGF10F10, FGF10F100, and FGF10F1000 for periods of up to 168 h (*n* = 3, average ± SD).

The coated sponges showed different bFGF-release profiles in a physiological salt solution depending on the fluoride concentration in the CaP solution. Figure 8 shows the change in bFGF concentration (measured by enzyme-linked immunosorbent assay (ELISA)) in the physiological salt solution during the incubation of FGF10F0, FGF10F10, FGF10F100, and FGF10F1000 for periods of up to 168 h (7 d). For all of the sponges, the bFGF concentration in the salt solution was highest at the initial sampling point at 3 h and decreased thereafter. The decrease in the bFGF concentration with time is due to the low stability of bFGF [21,35,36,37]. According to one study, bFGF added to an acellular culture medium at a concentration of 10 ng/mL became hardly detectable by ELISA within 1 day [37]. At all sampling points from 3 to 168 h, the bFGF concentration in the salt solution decreased basically in the following order: FGF10F0 > FGF10F10 > FGF10F100 > FGF10F1000.

### 2.5. Solubility Assay of the Coating Layers

**Figure 9:**
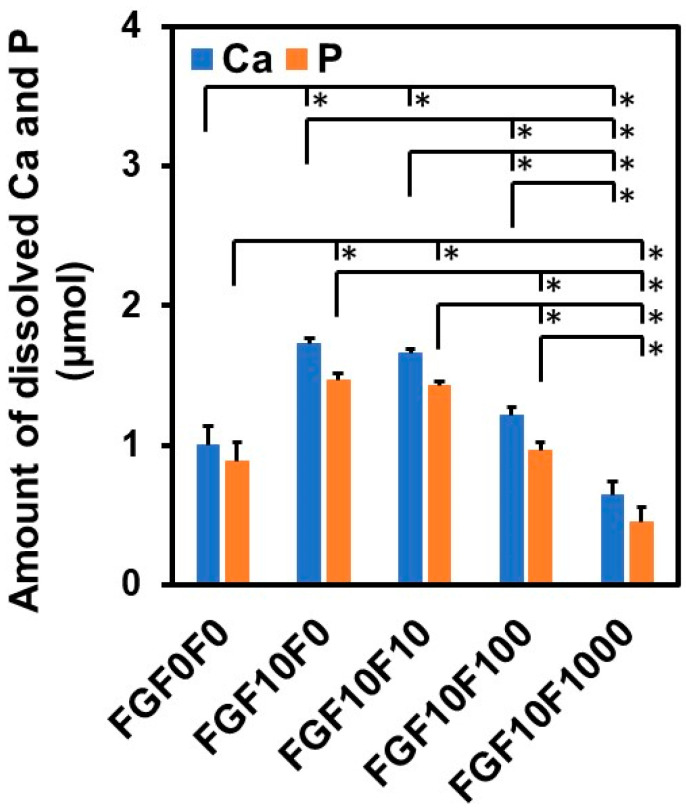
Amounts of Ca and P released from FGF0F0, FGF10F0, FGF10F10, FGF10F100, and FGF10F1000 after incubation for 7 d in the physiological salt solution (*n* = 3, average + SD, * *p* < 0.05).

The solubility of the coating layers decreased with the increasing fluoride concentration in the CaP solution from 10 to 1000 µM. In order to compare the solubility of the coating layers, the amounts of Ca and P that had been released from the sponges were measured at the endpoint (7 d) of the bFGF-release assay in the physiological salt solution by inductively coupled plasma (ICP) optical emission spectrometry. As shown in Figure 9, the amounts of Ca and P released from FGF10F0 were higher than those released from FGF0F0 and comparable to those released from FGF10F10. The amounts of Ca and P released into the salt solution decreased in the following order: FGF10F10 > FGF10F100 > FGF10F1000. The release of Ca and P from the coated sponges should be due to the partial dissolution of the coating layers in the salt solution.

### 2.6. Fluoride Ion Release in the bFGF-Release Assay

**Figure 10:**
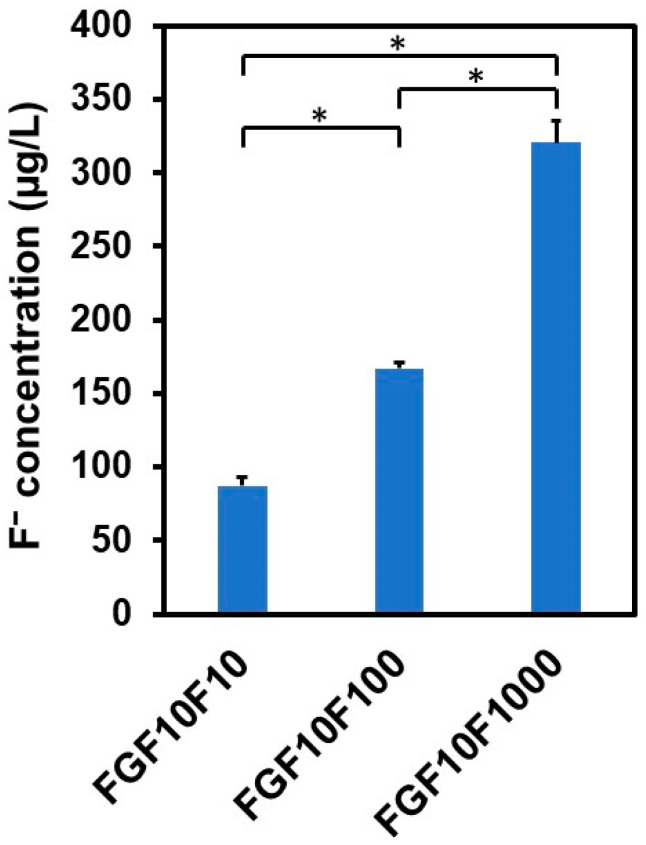
Concentration of fluoride ions in the physiological salt solution after incubation of FGF10F10, FGF10F100, and FGF10F1000 for 7 d (*n* = 3, average + SD, * *p* < 0.05).

The coated sponges released not only Ca and P, but also fluoride ions, into the physiological salt solution in the bFGF-release assay. As shown in Figure 10, the fluoride ion concentration in the salt solution at the endpoint (7 d) of the bFGF-release assay increased in the following order: FGF10F10 < FGF10F100 < FGF10F1000. These results indicate that fluoride ions were incorporated into the surface layers of these sponges, although no clear F peak was detected in the SEM-EDX spectrum of FGF10F10 (Figure 3b). Taking all of the surface characterization results into consideration, the coating layers on FGF10F10, as well as FGF10F100 and FGF10F1000, all appeared to be composed of FAp.

### 2.7. Mineralization Assay in a Simulated Body Fluid (SBF)

**Figure 11:**
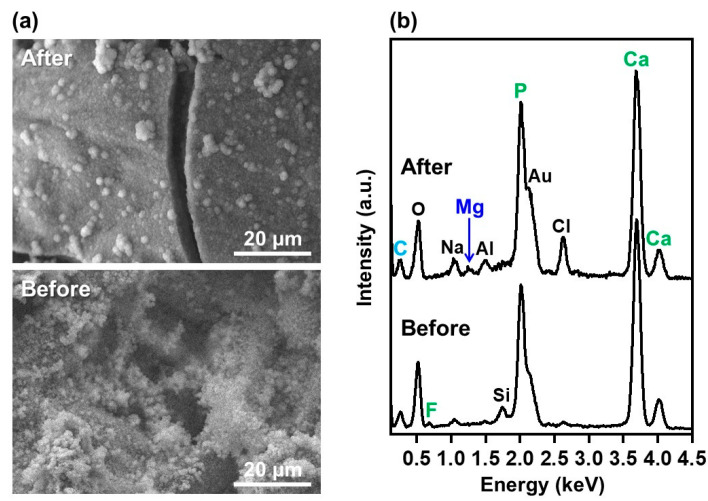
(**a**) SEM images and (**b**) SEM-EDX spectra of the outer surfaces of FGF10F1000 before and after immersion in SBF for 4 d. The peaks of Al and Au in (**b**) are attributed to an aluminum sample holder and a conductive coating for observation, respectively.

The mineralization potential of the coated sponge was suggested by the SBF test. The selected sponge (FGF10F1000) was immersed in SBF for 4 d, according to ISO23317 [38,39]. As shown in Figure 11a, a dense micro-thick layer was observed on the sponge after immersion in SBF. After immersion in SBF, Mg (element contained in SBF) was newly detected by SEM-EDX, whereas F (from FAp) became undetected (Figure 11b). From these results and studies [38,39], the micro-thick layer observed on the sponge should be a bone-like apatite layer grown in SBF.

## 3. Discussion

**Figure 12:**
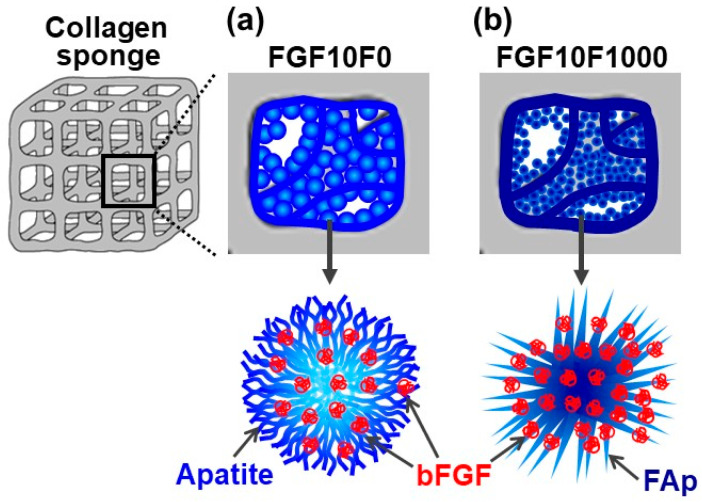
Schematic illustration of putative structures of (**a**) bFGF-immobilized apatite coating on FGF10F0 and (**b**) bFGF-immobilized FAp coating on FGF10F1000.

Using the present precursor-assisted biomimetic process, 3D porous collagen sponges were successfully coated on the outer and inner surfaces with bFGF-immobilized FAp layers with differing fluoride content. According to the experimental results, the coating layers formed on FGF10F10, FGF10F100, and FGF10F1000 were bFGF-immobilized FAp layers, whereas the coating layers formed on FGF0F0 and FGF10F0 were an apatite layer and a bFGF-immobilized apatite layer, respectively. Considering a previous study of a laminin-immobilized apatite layer [40] and the high protein-adsorption capability of apatite crystals [41,42], bFGF adsorbed onto not only the porous collagen surface, but also the apatite and FAp crystals, as illustrated in Figure 12. In the previous study [40], a larger (~7 times) amount of laminin was immobilized on a polymer plate by a similar biomimetic process using a laminin-containing CaP solution than by a simple adsorption process onto an apatite-precoated polymer plate using laminin-containing saline. On the other hand, from the shift of the apatite 130 diffraction peak (Figure 4a) and homogenous fluoride distribution (Figure 7c), fluoride ions should be incorporated into the crystal lattice of FAp via partial ionic substitution for hydroxyl ions of apatite, as reported elsewhere [28,29,43,44]. As expected, the fluoride content in the FAp layer (regarded as proportional to the degree of fluoride substitution) increased with the increasing fluoride concentration in the CaP solution from 10 to 1000 µM (FGF10F10 < FGF10F100 < FGF10F1000) (Figure 3b and Figure 4a). According to the F/P molar ratio (from STEM-EDX), the degree of fluoride substitution was approximately 40% for FGF10F1000. The mineralization potential of this sponge was suggested by the SBF test (Figure 11). This is not surprising, because SBF is supersaturated with respect to apatite, and the FAp layer coated on the sponge can grow spontaneously in SBF. Interestingly, the amount of bFGF immobilized in FGF10F1000 was higher than that in FGF10F0 (Figure 5), although the sponges were prepared in CaP solutions with the same bFGF concentration (10 µg/mL). This discrepancy could have occurred because the FAp crystals grew faster in the CaP solutions in the presence of fluoride ions and provided more adsorption sites for bFGF than the apatite crystals, owing to the lower aqueous solubility of FAp [29,45,46]. This possibility is supported by the TEM and XRD analyses, in which FGF10F1000 showed more developed and matured FAp crystals with a higher Ca/P ratio (see Section 2.3) and more-intense diffraction peaks of FAp (Figure 4a) compared to FGF10F0. In addition, FGF10F1000 gained more coating weight than FGF10F0 (Table 1).

The tailored bFGF release was achieved by incorporating fluoride ions into the coating layer. As demonstrated in Figure 8, the coated sponges released different concentrations of bFGF (from 1 to 10 ng/mL at 3 h) in the physiological salt solution, depending on their fluoride content. Despite the higher bFGF content, FGF10F1000, with a higher fluoride content, released a lower concentration of bFGF than FGF10F0 without fluoride. The coating layers on FGF10F100 and FGF10F1000 maintained the bFGF concentration in the salt solution in a narrower range throughout the assay period (from 3 to 168 h), without causing a significant initial burst. The tailored release of bFGF from the coated sponge can be attributed to the solubility control of the coating layer due to fluoride incorporation. The difference in the coating weight (correlated to the coating thickness) might also be involved in the tailored bFGF release. The negative correlation between the fluoride content and the solubility of apatite crystals has been reported elsewhere [28,46,47]. The reduced solubility of the coating layer due to fluoride incorporation was verified by the result of ICP analysis, demonstrating that the amounts of Ca and P dissolved from the coated sponge in the physiological salt solution decreased in the following order: FGF10F0, FGF10F10 > FGF10F100 > FGF10F1000 (Figure 9). Note that the solubility of the bFGF-immobilized apatite layer on FGF10F0 was higher than that of the apatite layer on FGF0F0 (neither of which contained fluoride). This might have been due to the inhibitory effect of bFGF on the growth and maturation of the apatite crystals in the CaP solution, as reported for some other biomolecules [40].

In the bFGF-release assay described above, ELISA was employed to detect immunoreactive bFGF (bFGF that retains its binding sites for a monoclonal antibody specific to human bFGF) released from the coated sponges. As shown in Figure 8, the concentrations of ELISA-detectable bFGF in the release assay were in the order of nanogram, whereas the amounts of bFGF immobilized on the sponges, quantified by the Bradford protein assay, were in the order of microgram (Figure 5). This discrepancy is not surprising considering the extremely low stability (immunoreactivity) of bFGF, even under physiological conditions [21,35,36,37]. For example, a previous study showed that apatite layers immobilizing 1.5 ± 0.6 and 1.6 ± 0.4 μg of bFGF (quantified by the Bradford protein assay) released 39.09 ± 12.45 pg and 89.47 ± 33.51 pg of bFGF (quantified by ELISA), respectively, in a medium (1 mL) after incubation for 3 d [24].

It is known that bFGF exerts its biological activity even at nanogram levels, with a bell-shaped dose-dependency; moreover, there is an optimum bFGF dose, and overdosing may cause adverse effects [25,48,49]. For example, bone morphogenetic protein-2 (BMP-2) expression in MG-63 cells (osteoblasts) increases with increasing bFGF concentration in a medium up to 10 ng/mL and decreases at 30 ng/mL [25]. Therefore, it is highly important to control the local concentration of bFGF in the body to maximize its osteogenic potential. The bFGF concentrations derived from the coated sponges were comparable to the reported effective bFGF levels for the promotion of bone regeneration [25] and angiogenesis [26]. The proposed approach for the tailored release of bFGF using the FAp coating layer would be useful for the construction of a mineralized, bFGF-releasing collagen scaffold that is highly beneficial for bone tissue engineering. Furthermore, use of the FAp layer as an immobilization matrix for bFGF may add additional value to the scaffold due to the co-release of fluoride ions. As shown in Figure 10, fluoride ions, along with bFGF, were released from the coated sponges in the physiological salt solution. The resulting fluoride concentrations (87 µg/L, 167 µg/L, and 321 µg/L, on average, for FGF10F10, FGF10F100 and FGF10F1000, respectively) were higher than the concentrations in human body fluids (8.5–45 µg/L) [50], much lower than the cytotoxic level for human oral mucosal fibroblasts [51], and comparable to the effective concentration for eliciting osteogenic activity in vitro [52].

This study has limitations, as described below. Although this study provided a coating strategy for tailored bFGF release from a collagen sponge, the cellular responses to the coated sponges are yet to be elucidated. In vitro studies using osteogenic and osteoclastic cells are needed in the future to evaluate cell–material interactions. Second, bFGF release was assayed in a physiological salt solution (pH = 7.4) that is different from actual in vivo conditions. In general, a scaffold is temporally exposed to an acidified environment after implantation resulting from postoperative inflammation reactions [53]. It is known that both apatite and FAp crystals show increased solubility in an acidified environment compared to that found in a normal physiological environment [47,54]. Thus, bFGF release (Ca and P release as well) from the coated sponges should be enhanced temporally in an early post-implantation stage (acidified environment) before the subsequent healing stage (neutral environment). Release control of bFGF under an acidified environment can be a topic for future study. Furthermore, in vivo studies using animals with critical size bone defects are needed to investigate the biological properties (biocompatibility, mineralization properties, osteogenic potential, bioresorbability, etc.) of the coated sponges and to create a scaffold with a formulation optimized for bone tissue engineering.

## 4. Materials and Methods

### 4.1. Materials

A sterile sponge sheet of calf-dermal-derived type I and III atelo-collagen (Terudermis) was purchased from GC corporation, Tokyo, Japan. Fiblast^®^ (Kaken Pharmaceutical Co., Ltd., Tokyo, Japan) was used as the bFGF source. All other reagents, as described in Section 4.2, Section 4.3, Section 4.7, Section 4.8 and Section 4.9, were reagent-grade chemicals obtained from Nacalai Tesque, Inc., Kyoto, Japan, unless otherwise specified.

### 4.2. Preparation of Supersaturated CaP Solutions

Five supersaturated CaP solutions with differing bFGF and fluoride concentrations were prepared, as shown in Table 2. First, a mother CaP solution containing neither bFGF nor fluoride was prepared as per the previous protocol [5]. Briefly, NaCl (final concentration = 142 mM), K_2_HPO_4_·3H_2_O (1.50 mM), HCl (40 mM), and CaCl_2_ (3.75 mM) were dissolved in ultrapure water, and the solution was buffered to pH 7.40 at 25 °C with tris(hydroxymethyl)aminomethane (TRIS, final concentration = 50 mM) and the necessary amount of 1 M HCl. The mother CaP solution was supersaturated with respect to hydroxyapatite (supersaturation = 19.9) and octacalcium phosphate (supersaturation = 2.06) [55]. Despite such high supersaturation, the mother CaP solution remained stable at around 5 °C for 4 weeks without inducing homogeneous precipitation. Thus, the mother CaP solution was stored in a sealed container at 5 °C and used within 4 weeks of preparation.

Just prior to the precursor-assisted biomimetic process (Section 4.3), the mother CaP solution was supplemented with 10 µg/mL bFGF. In this fluoride-free solution, NaF (1000 μM) was dissolved under stirring conditions for 30 min. The resulting solution was diluted with the fluoride-free solution to prepare supersaturated CaP solutions with lower fluoride concentrations (10 µM and 100 μM) and the same bFGF concentration (10 µg/mL). All solutions were transparent without any precipitation and filtered using a 0.22-µm-pore-sized filter before use.

### 4.3. Precursor-Assisted Biomimetic Process for Coating

**Figure 13:**
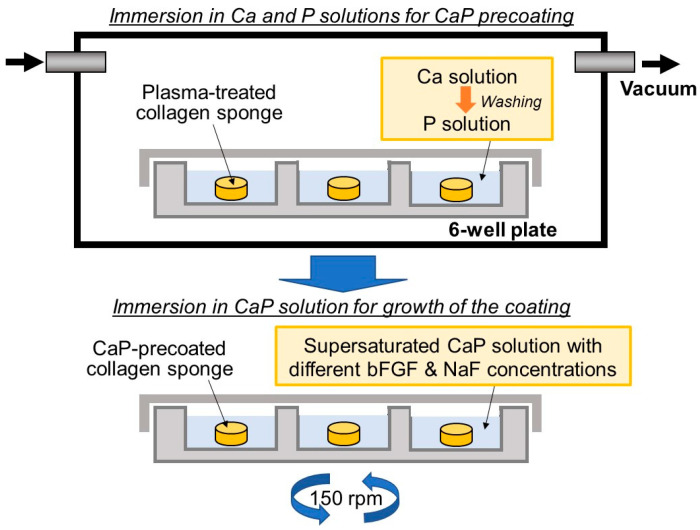
Schematic illustration of the precursor-assisted biomimetic process for preparing collagen sponges: (**upper**) sequential immersion in calcium and phosphate solutions for CaP precoating and (**lower**) subsequent immersion in supersaturated CaP solution with different bFGF and NaF concentrations for the growth of the coating layer.

The collagen sponges were prepared by cutting the 3-mm-thick collagen sheet into circular disks 5 mm in diameter using an SD-lever-type sample cutting machine (SDL-200, DUMBBELL Co., Ltd., Kawagoe, Japan). The collagen sponges were subjected to the precursor-assisted biomimetic process under aseptic conditions, as previously described [5], with slight modifications (Figure 13).

The collagen sponges were subjected to oxygen plasma treatment for 2 min in O_2_ gas (30 Pa) at a power density of 0.1 W/cm^2^ under an electric field operated at 13.56 MHz using a compact ion etcher (FA-1, Samco Inc., Kyoto, Japan). The plasma-treated sponges were precoated with CaP by sequential immersion in calcium and phosphate solutions, as described below (upper schematic in Figure 13). First, each plasma-treated sponge was immersed in 10 mL of a 200 mM CaCl_2_ aqueous solution (in a 6-well plate; the same applies below) for 15 min under reduced pressure using a vacuum desiccator. The same immersion process was performed using a 200 mM K_2_HPO_4_·3H_2_O solution after washing the sponge with 10 mL of ultrapure water.

After removal from the phosphate solution, the CaP-precoated sponges were subsequently immersed in 10 mL of the CaP solution (prepared as described in Section 4.2) for solution displacement and then immersed in 10 mL of the fresh CaP solution. The sponges in the CaP solutions were incubated at 25 °C for 24 h with shaking at 150 rpm using a shaking incubator (M-BR-104P, TAITEC CORPORATION, Koshigaya, Japan) for the growth of the coating layer (lower schematic in Figure 13). After incubation for 24 h, the sponges removed from the CaP solution were washed with ultrapure water, frozen at −80 °C, and lyophilized using a freeze-drier (FDS-1000, TOKYO RIKAKIKAI CO., LTD., Tokyo, Japan). The lyophilized sponges were kept at −30 °C before further use.

To assess the amount of bFGF immobilized on the sponges (Section 4.4), the CaP solutions without sponges (Control Solution A) and a 142 mM NaCl solution (buffered to pH 7.40 at 25 °C using TRIS (50 mM) and the necessary amount of 1M HCl) containing the untreated sponge (Control Solution B) were also incubated for 24 h under the same conditions (25 °C, 150 rpm).

### 4.4. Assessment of Coating Weight and bFGF Content in Coated Sponges

The weight measurements were performed for the untreated sponges and those after coating using an analytical balance (GR-202, A&D Company, Ltd., Tokyo, Japan), with a minimum weight of 0.01 mg, to estimate the coating weight of each sponge.

After incubation for 24 h in the shaking incubator (Section 4.3), the CaP solutions and Control Solutions A and B were subjected to the protein assay using a Sigma-Aldrich Bradford reagent (Merck KGaA, Darmstadt, Germany) according to the manufacturer’s instructions. The amount of residual bFGF in the CaP solution was estimated by subtracting the amount of protein in Control Solution B from that in the CaP solution. The amount of bFGF immobilized on each sponge was estimated by subtracting the residual bFGF in the CaP solution from that in Control Solution A (i.e., the corresponding CaP solution without a sponge).

### 4.5. Surface Characterization

A digital image of an untreated collagen sponge was captured using a digital camera (Tough TG-6, OM Digital Solutions Corporation, Hachioji, Japan). The sponge was cut in half horizontally with microtome blade (S-35, Feather Safety Razor Co., Ltd., Osaka, Japan) to expose its inner surface. The sponges were mounted on an aluminum sample holder and sputter-coated with gold for 2 min using a sputter-coating machine (SC-701MkII, Sanyu Electron Co., Ltd., Akishima, Japan) to ensure electrical conductivity. The outer and inner surfaces of the untreated and coated sponges were analyzed using an SEM (TM4000Plus II, Hitachi High-Tech Corp., Tokyo, Japan) equipped with an EDX spectrometer (AZtecOne, Oxford Instruments plc, Abingdon, UK). In the SEM analysis, the accelerating voltage and working distance were set at 15 kV and 7–12 mm, respectively, and the scanning time (live time scan) was set at 120 s for EDX analysis.

The outer surfaces of the sponges were examined using an XRD analyzer (M18X, MacScience, Yokohama, Japan) with CuKα radiation (λ = 0.154178 nm) and an FT-IR spectrometer (FT/IR-4700, JASCO Corporation, Hachioji, Japan) equipped with an attenuated total reflection accessory and monolithic diamond crystal. The XRD analyzer was operated under the fixed power of 8 kW (40 kV, 200 mA). The FT-IR measurements were carried out in the range of 600–4000 cm^−1^ with a scan number of 80.

For selected sponges (uncoated, FGF10F0, and FGF10F1000), the outer surfaces were analyzed using a 3D laser scanning confocal microscope (VK-X3050, Keyence Corporation, Osaka, Japan) with the laser light at 661 nm to measure their 2D porosity (percentage of the void (concave) area among the analyzed area). In the measurements, an objective lens of ×5 (total magnification of ×120) was used to analyze the surface area of 2.2 mm × 3.0 mm, with a working distance of 22.5 mm.

### 4.6. Ultrastructural Analysis

The nanostructure and elemental distribution of the coatings formed on the selected sponges (FGF10F0 and FGF10F1000) were further investigated using an analytical TEM (FEI Tecnai Osiris, FEI, Hillsboro, OR, USA) operated at 200 kV equipped with an EDX spectrometer (Super-X system, FEI, USA) and a HAADF-STEM system with an electron probe smaller than 1 nm in diameter. Prior to the TEM analyses, the coating layer was scraped from the sponge, ultrasonically dispersed in ethanol, and mounted on a Cu grid covered with holey carbon film.

### 4.7. bFGF-Release Assay

The release of bFGF from the coated sponges was examined in the physiological salt solution. The physiological salt solution was prepared by dissolving NaCl (final concentration = 142 mM) in ultrapure water and buffering to pH 7.4 at 37 °C using TRIS (50 mM) and the necessary amount of 1 M HCl. Each of the sponges was immersed in 5.0 mL of the salt solution and degassed for approximately 30 min in a vacuum desiccator. The sponge submerged in the salt solution was then incubated at 37 °C for up to 168 h (7 d). After incubation for 0, 3, 24, 72, and 168 h, an aliquot of 100 µL was sampled from the salt solution and stored at −80 °C before ELISA. The sampled solutions were diluted and assayed for bFGF using a human FGF basic Quantikine^®^ ELISA kit (R&D Systems, Inc., Minneapolis, MN, USA) according to the manufacturer’s instructions.

### 4.8. Quantification of the Released Ca and P Atoms and Fluoride Ions

After incubation for 168 h (7 d) in the bFGF-release assay (Section 4.7), Ca and P atoms and fluoride ions in the residual salt solutions were quantified. Ca and P in the residual salt solutions were quantified using an ICP optical emission spectrometer (ULTIMA2, HORIBA, Ltd., Kyoto, Japan) after 10-fold dilution with 0.1 M HCl aqueous solution. The concentration of fluoride ions in the residual salt solutions was measured using a fluoride-ion-selective electrode (6561S-10C, HORIBA, Ltd., Japan) with a pH/ORP/ion meter (D-73, HORIBA, Ltd.) after 3-fold dilution with the physiological salt solution.

### 4.9. SBF Test

The SBF test was performed for the selected sponge (FGF10F1000) according to ISO23317. SBF (the following so-called c-SBF: 142.0 mM Na^+^, 5.0 mM K^+^, 1.5 mM Mg^2+^, 2.5 mM Ca^2+^, 147.8 mM Cl^−^, 4.2 mM HCO_3_^−^, 1.0 mM HPO_4_^2−^, and 0.5 mM SO_4_^2−^) was prepared by dissolving NaCl, NaHCO_3_, KCl, K_2_HPO_4_·3H_2_O, MgCl_2_·6H_2_O, HCl (aq), CaCl_2_, and Na_2_SO_4_ into ultrapure water and buffering the solution to pH 7.40 at 36.5 °C using TRIS (50 mM) and the necessary amount of 1 M HCl, as per the protocol described elsewhere [38,39]. The sponge was pre-immersed in 10 mL of SBF and degassed for 30 min in a vacuum desiccator. Afterward, the sponge was immersed in 200 mL of fresh SBF and incubated at 36.5 °C for 4 d. After immersion for 4 d, the sponge was removed from SBF, washed thrice with ultrapure water, and freeze-dried for SEM-EDX analyses. SEM-EDX analyses were performed using the same method described in Section 4.5.

### 4.10. Statistical Analysis

For quantitative analyses, 4 analytical regions (for STEM-EDX analysis) or 3 sponges (for all other analyses) were used for each condition to obtain an average value and SD. Student’s *t*-test was used to determine the significant differences between two groups. A single-factor analysis of variance (ANOVA) was used to identify significant differences among three or more groups. When significant differences were observed, Tukey’s post hoc multiple-comparison test was used to determine the differences between the average values of the groups. The statistical significance was set at *p* < 0.05.

## 5. Conclusions

Using the precursor-assisted biomimetic process, bFGF-immobilized FAp layers with varying fluoride contents were coated on the outer and inner surfaces of the 3D porous collagen sponge. The fluoride ions incorporated in the apatite crystals affected the solubility of the coating layer, thereby enabling the tailored release of bFGF from the coated sponges into the physiological salt solution. The present coating process would be useful for the construction of a mineralized, bFGF-releasing collagen scaffold beneficial for bone tissue engineering.

## Figures and Tables

**Table 1:** The 2D porosity (measured by laser microscopy) of the untreated sponge, FGF10F10, and FGF10F1000, and coating weight and Ca/P and F/P molar ratios (measured by STEM-EDX) of the coatings in FGF10F10 and FGF10F1000 (*n* = 4 for molar ratios and 3 for the others, average ± SD).

	Untreated	FGF10F0	FGF10F1000
2D porosity (%)	58 ± 10	44 ± 6	38 ± 6
Coating weight (mg/mg of sponge)	−	2.08 ± 0.09	2.68 ± 0.22
Ca/P molar ratio of the coating	−	1.54 ± 0.02	1.65 ± 0.03
F/P molar ratio of the coating	−	−	0.14 ± 0.04

**Table 2:** Concentrations of bFGF and NaF in the supersaturated CaP solutions used for coating and the names of the resulting coated collagen sponges.

bFGF (μg/mL)	NaF (μM)	Sample Name
0	0	FGF0F0
10	0	FGF10F0
10	10	FGF10F10
10	100	FGF10F100
10	1000	FGF10F1000

## Data Availability

Data are contained within the article.
